# 627. Outcomes of Military Blood Donors Screening Positive for *Trypanosoma cruzi*

**DOI:** 10.1093/ofid/ofae631.192

**Published:** 2025-01-29

**Authors:** Michael Wells, Brian Casleton, Glorimar Rivera, Megan Phelps, Theresa Casey, Angela Osuna, Melita Gella, Ga O Jung, Erin Winkler, Heather Yun, Joseph Marcus

**Affiliations:** Brooke Army Medical Center, Cibolo, TX; Armed Services Blood Bank San Antonio, San Antonio, Texas; Armed Services Blood Bank San Antonio, San Antonio, Texas; DHA Lackland 59th MDW, San Antonio, Texas; BAMC, San Antonio, Texas; BAMC, San Antonio, Texas; Armed Services Blood Bank San Antonio, San Antonio, Texas; 559th Medical Group, JBSA-Lackland, Texas; BAMC, San Antonio, Texas; Brooke Army Medical Center, Cibolo, TX; Brooke Army Medical Center, Cibolo, TX

## Abstract

**Background:**

First time blood donors at the Armed Service Blood Bank-San Antonio are screened for *Trypanosoma cruzi* infection with a chemiluminescent microparticle immunoassay (CMIA) followed by a confirmatory enzyme strip assay (ESA). In military populations, *T. cruzi* positivity on a screening test accounts for the fourth most common reason for post-donation deferral. With concerns for low positive predictive value in a low prevalence disease, this study evaluated the clinical outcomes and diagnostic work-up of blood donors who screened positive on *T. cruzi* CMIA testing.

**Table 1:**
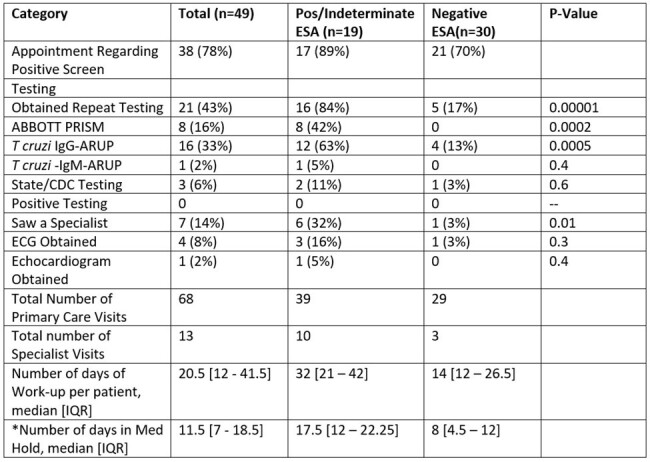
Workup of 49 blood donors who screened positive for Trypanosoma cruzi infection

**Methods:**

All blood donors from 2017-2022 were screened for *T. cruzi* antibodies with CMIA and deferred indefinitely if screening was positive, regardless of ESA result. All donors that initially screened positive for *T. cruzi* infection were evaluated to determine diagnostic work-up, further testing, specialty appointments seen, and final diagnosis of each case following their initial positive blood donation screen.

**Table 2:**
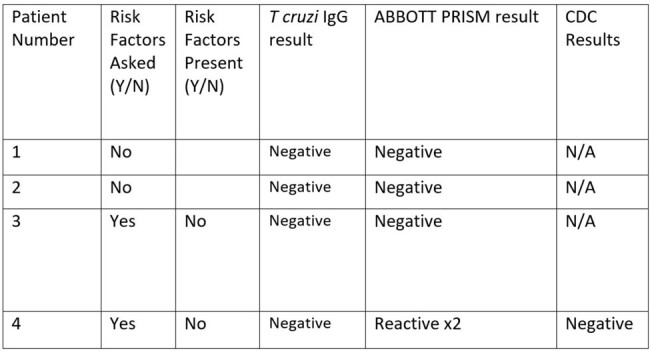
Clinical outcomes of 4 blood donors with positive T. cruzi screening test and confirmatory test

**Results:**

Of 89,459 blood donors during the study period, 49 (57.3 per 100k donors) screened positive for Chagas disease on initial blood donation. Of those individuals, 4 (8%) had positive, 18 (36%) had indeterminate, and 30 (61%) had negative confirmatory tests. The evaluation varied significantly depending on the results of ESA testing, however there were no blood donors who met criteria for Chagas disease (**Table 1**). Evaluation of four donors with positive CMIA and ESA showed only one with repeat positivity on one assay, but all were ultimately deemed negative (**Table 2)**.

**Conclusion:**

While positive *T. cruzi* antibodies were a common reason for post-donation deferral in this cohort, there were no confirmed cases of Chagas disease, despite most patients receiving an extensive work-up. Patients with positive or indeterminate ESA received more specialty care and more confirmatory testing than those with a negative confirmatory test. This study demonstrates the need for higher specificity screening and confirmatory tests for *T. cruzi* in blood donors in low prevalence settings.

**Disclosures:**

**All Authors**: No reported disclosures

